# The Normal Diet

**Published:** 1890-02

**Authors:** 


THE "NORMAL" DIET.--Mr. G. Munro Smith, writing on this subject, states that the daily destructive metabolism, which is the great criterion of work done, does not vary much amongst different occupations. Premising that he does not consider moderate over-eating injurious, he finds that very many men eat considerably more than the most liberal tables; it is not an uncommon thing for an average sized man on very moderate work to eat twenty-five or twenty-seven ounces of chemically dry food a day. Women eat much less than men, after making allowances for differences in weight and work. Where a man eats nineteen ounces, a woman of the same weight and of active habits eats only fourteen or fifteen ounces. On a diet from which all meat is excluded, he has found that twelve to thirteen ounces *per diem* will comfortably feed a hard-working man. A moderate amount of stimulants appears to increase the average; moderately free drinking diminishes it. A diet consisting of one part of nitrogenous to seven or eight non-nitrogenous is a good combination; it is greatly exceeded on the nitrogenous side by the majority of men and women, especially the former. A diet of twelve to fourteen ounces of chemically dry food, digestible, with the ingredients in proper proportion, is sufficient to keep in good health an average sized man on moderate work. The majority of people in England eat literally twice as much as this.—*British Med.-Chir. Journal.*.

